# Describing Diet of Imperiled Sierra Nevada Red Foxes and a Carnivoran Competitor Using DNA Metabarcoding

**DOI:** 10.1002/ece3.71605

**Published:** 2025-06-30

**Authors:** Matthew S. Delheimer, Marie E. Martin, Jennifer Hartman, Katie M. Moriarty, Jennifer M. Allen, Taal Levi

**Affiliations:** ^1^ USDA Forest Service, Tahoe National Forest Sierraville California USA; ^2^ Institute for Natural Resources Oregon State University Corvallis Oregon USA; ^3^ Conservation Canines University of Washington Seattle Washington USA; ^4^ Rogue Detection Teams Rice Washington USA; ^5^ National Council for Air and Stream Improvement, Inc. Corvallis Oregon USA; ^6^ Department of Fisheries, Wildlife, and Conservation Science Oregon State University Corvallis Oregon USA

## Abstract

Montane red foxes (*Vulpes vulpes* ssp.) native to western North America are of broad conservation interest, occupying a narrow ecological niche and typically restricted to small, isolated populations. However, many aspects of montane red fox ecology are poorly understood due to their rarity. We examined Sierra Nevada red fox (*V. v. necator*) diet in an unstudied portion of their range, then evaluated dietary overlap with coyote (*Canis latrans*), a presumably dominant carnivore expected to exhibit future distributional increases. We collected Sierra Nevada red fox and coyote scats via detection dog team surveys during 2017–2018 in Oregon, USA and used DNA metabarcoding to determine scat composition. Sierra Nevada red fox and coyote diets differed with respect to the most frequently occurring prey species and prey species that comprised the largest proportions of their diets (red fox – golden‐mantled ground squirrel [*Callospermophilus lateralis*]; coyote – snowshoe hare [*Lepus americanus*]). Despite some differences, Sierra Nevada red fox and coyote diets exhibited similar taxonomic richness and their dietary overlap was high (Pianka's index = 0.74 via weighted percent occurrence, 0.69 via frequency of occurrence), with golden‐mantled ground squirrel appearing to be an important prey item for both species. High dietary overlap suggests potential for competition between Sierra Nevada red foxes and coyotes where the two species occur sympatrically, which could be consequential for foxes in the future if spatial overlap increases and results in niche compression. Our work addresses an aspect of data insufficiency for an imperiled species that can inform conservation strategies and species management.

## Graphical abstract

Montane red foxes ( *Vulpes vulpes* ssp.) native to western North America are of broad conservation interest, but their ecology is poorly understood due to their rarity. We examined the diet of the Sierra Nevada red fox (*V. v. necator*) in an unstudied portion of their range in Oregon, USA, then evaluated dietary overlap between red fox and coyote ( *Canis latrans* ), a presumably dominant carnivore. High dietary overlap suggests potential for competition between Sierra Nevada red foxes and coyotes where the two species occur sympatrically, which could be consequential for foxes in the future if spatial overlap increases and results in niche compression.

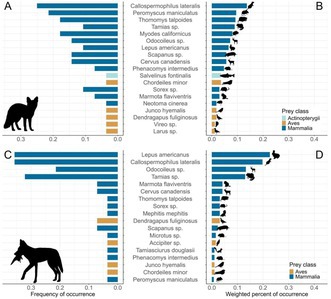

## 1 Introduction

The red fox ( *Vulpes vulpes* ) is one of the most widely distributed and well‐studied terrestrial carnivores globally (e.g., Devenish‐Nelson et al. 2013; Main et al. 2020; Castañeda et al. 2022). In stark contrast, montane red foxes native to western North America (Cascade [*V. v. cascadensis*], Rocky Mountain [*V. v. macroura*], and Sierra Nevada [*V. v. necator*] subspecies) are rare and poorly understood (Kamler and Ballard 2002; Perrine et al. 2010; Lewis et al. 2022). Montane red foxes occupy a narrow ecological niche, occurring in high‐elevation subalpine and alpine habitats of mountainous regions, with climates characterized by cold winter temperatures and deep snowpacks (Aubry et al. 2009; Quinn et al. 2018). Montane red foxes are genetically and morphologically distinct from their lower‐elevation counterparts in North America, which include both indigenous red foxes and nonnative red foxes originating from fur farms (Aubry et al. 2009; Sacks et al. 2010; Quinn et al. 2022). Populations of Cascade and Sierra Nevada red foxes are exceedingly small across their ranges in the Pacific states (Quinn et al. 2022; Green et al. 2023), which occur in Washington (Cascade red fox) and California and Oregon (Sierra Nevada red fox), respectively. Accordingly, both subspecies are of regional or national conservation concern—the Sierra Nevada red fox is listed as state threatened in California (CDFG 1980) and state sensitive in Oregon (Oregon Conservation Strategy 2016), while a “Sierra Nevada distinct population segment” of the Sierra Nevada red fox was federally listed as endangered in the United States in 2021 (USFWS 2021). In Washington, the Cascade red fox is listed as state endangered (Lewis et al. 2022).

Many aspects of montane red fox ecology remain little‐studied (Perrine et al. 2010; Lewis et al. 2022; Sierra Nevada Red Fox Conservation Advisory Team 2022) despite the need for robust information in guiding effective conservation of at‐risk carnivores (Martin et al. 2022). For instance, diet is a fundamental component of carnivore life history and important to evaluating individual fitness, habitat use, and population dynamics (Fryxell et al. 1999; Gómez‐Ortiz et al. 2011; Petrunenko et al. 2016). Dietary breadth can indicate a species' capacity to occupy altered landscapes, such as an ability to exploit novel food items or switch to alternate prey when primary prey is scarce (Moss et al. 2016; Fleming and Bateman 2018; Karandikar et al. 2022). Globally, red foxes have one of the broadest ecological niches of any mammal and accordingly, their diets are diverse and can be highly variable between different locations (Castañeda et al. 2022). However, the narrow ecological niche occupied by montane red foxes could constrain their dietary niche or otherwise limit their adaptability to variation in food resources (e.g., fluctuations in prey abundance or reduced availability of a primary prey species). Dietary plasticity or diversity may therefore be advantageous for montane red foxes, yet few studies are available to evaluate their diet patterns (but see Aubry 1983; Perrine 2005; Cross and Crabtree 2021; Rosburg‐Francot et al. 2025).

Diet is also relevant to examining interactions (e.g., competition, predation, subsidization) that shape and structure carnivore communities (Lanszki et al. 2019; Prugh and Sivy 2020; Ruprecht et al. 2021). Although montane red foxes could experience competitive pressure from various similar‐sized sympatric carnivores (e.g., American badger [ *Taxidea taxus* ], bobcat [ *Lynx rufus* ], fisher [ *Pekania pennanti* ], Pacific marten [ *Martes caurina* ]), coyotes ( *Canis latrans* ) have been specifically posited as being impactful (Perrine et al. 2010; USFWS 2021; Lewis et al. 2022; Sierra Nevada Red Fox Conservation Advisory Team 2022). Coyotes (7–20 kg; Bekoff 1977) are larger‐bodied than montane red foxes (3.3–4.3 kg; Perrine et al. 2010) and are known predators of lower‐elevation red foxes and other fox species (Kamler et al. 2003; Farias et al. 2005; Gosselink et al. 2007). Coyotes are generalists exhibiting a high degree of behavioral and dietary plasticity (Breck et al. 2019; Petroelje et al. 2021; Webster et al. 2022), thus exploitative and nonlethal interference competition have also been widely indicated to occur between coyotes and low‐elevation foxes (Levi and Wilmers 2012; Newsome and Ripple 2015; Lonsinger et al. 2017; Masters and Maher 2022). Historically, coyote presence in areas inhabited by montane red foxes was thought to be limited to nonwinter seasons because coyote morphology is not well‐suited to moving through deep snow (Murray and Boutin 1991). However, coyotes will likely have greater access to these areas as snowpacks decrease in the future (e.g., Kolbe et al. 2007; Dowd et al. 2014). Changing conditions could modify the pathways by which subordinate carnivores such as montane red foxes have previously differentiated (e.g., spatial or resource niches) with dominant species (Manlick et al. 2017; Smith et al. 2018).

Interactions between carnivores are difficult to directly observe and thus the existence, strength, or direction of competitive relationships is often unclear, with some exceptions (e.g., intraguild predation; Fedriani et al. 2000). However, proxies such as dietary overlap between species can provide insights into the potential for competition—for instance, high dietary overlap between sympatric carnivores might indicate a greater likelihood of competitive interactions (Dröge et al. 2017). Here, we examined the diet of Sierra Nevada red fox and coyote in the Cascade Mountains of Oregon, using DNA metabarcoding to determine the composition of scats collected via detection dog team surveys. DNA metabarcoding is an emerging method of diet analysis that has proven invaluable to recent studies of carnivore diets (Eriksson et al. 2019; Hacker et al. 2021; Massey et al. 2021; McLennan et al. 2022; Tosa et al. 2023), while scat surveys offer a noninvasive method to collect biological samples that is effective for studying rare or elusive species (Rodgers and Janečka 2013; Boucher et al. 2019; Hacker et al. 2021; Moriarty et al. 2021). Our research objectives were to (1) describe the diet composition of Sierra Nevada red fox in an unstudied portion of montane red fox distribution (Oregon), and (2) evaluate dietary overlap between Sierra Nevada red foxes and coyotes. We compare our findings to the few studies of montane red fox diet that have occurred elsewhere in western North America and consider montane red fox diet in the context of changing conditions that could affect access to food resources or alter competitive interactions.

## 2 Materials and Methods

### 2.1 Study Area

**FIGURE 1:**
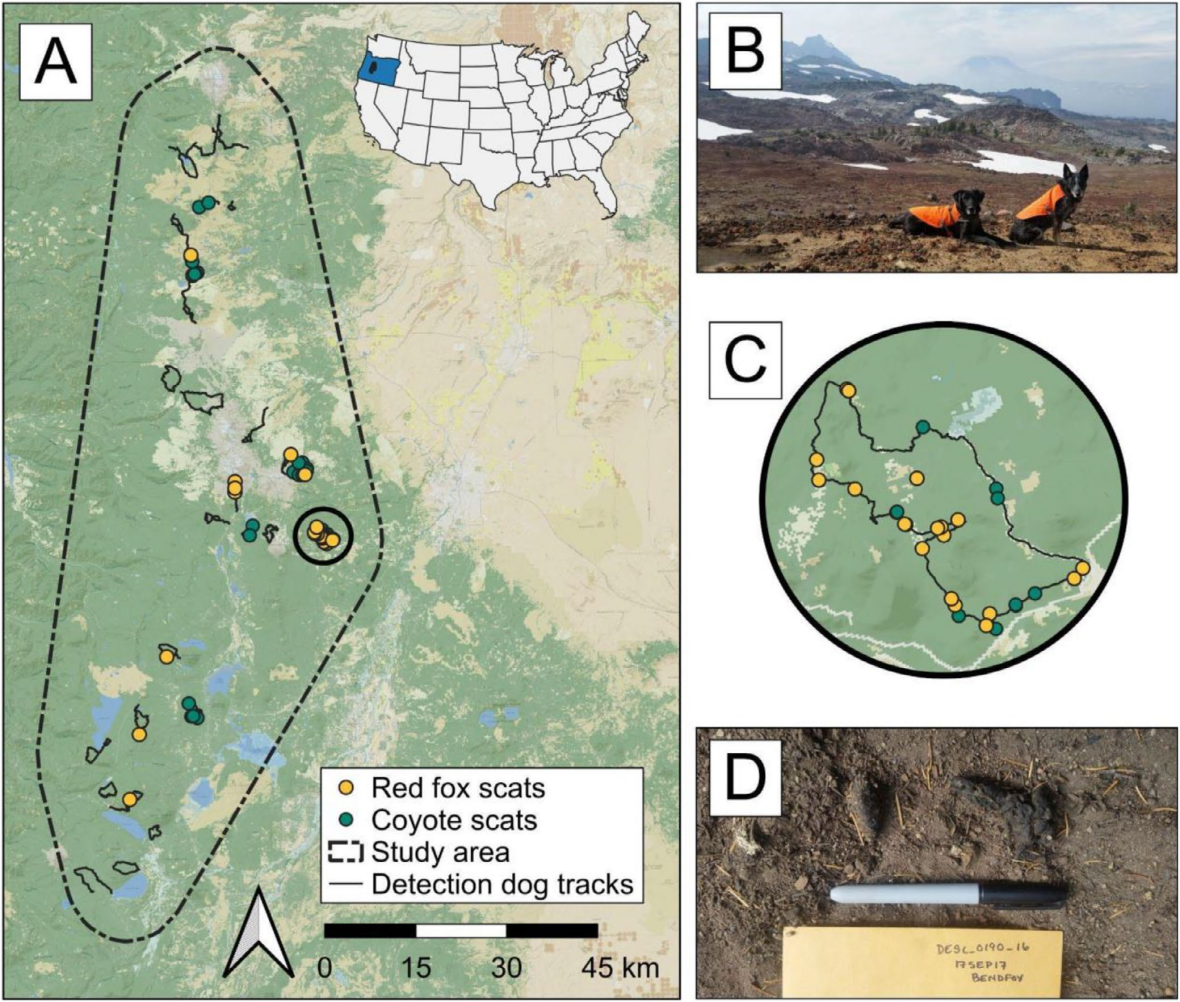
Study area (2773 km^2^) to collect scat samples from Sierra Nevada red foxes ( *Vulpes vulpes necator* ) and coyotes ( *Canis latrans* ) in the Cascade Mountains of Oregon, USA, during 2017 including: (A) all detection dog team survey routes (“tracks”) and scat collection locations; (B) example of a detection dog team and surveyed red fox habitat; (C) example of a survey route with scat collections; and (D) example of a collected red fox scat.

**FIGURE 2:**
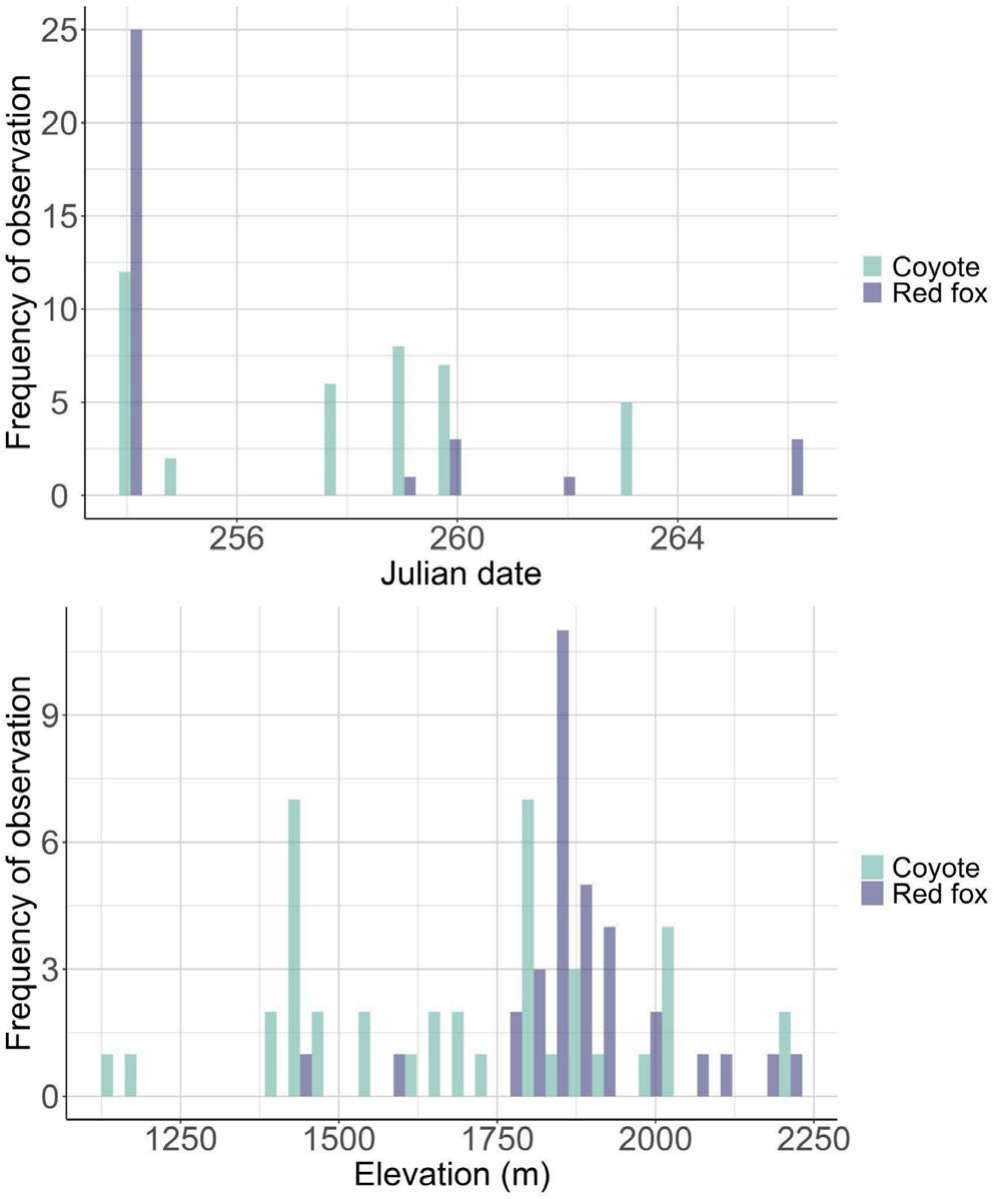
Collection dates and elevation profiles of Sierra Nevada red fox ( *Vulpes vulpes necator* ) and coyote ( *Canis latrans* ) scats collected during 2017 in the Cascade Mountains of Oregon, USA.

Our study occurred in western Oregon, USA, across 2773 km^2^ at elevations ranging from 1020 to 2463 m (Figure 1, Figure 2). The West Cascade ecoregion of Oregon is characterized by steep, forested terrain surrounding high‐elevation volcanic peaks of the Cascade Mountains, with nonforested features including bare rock, permanent snowfields, and alpine areas (e.g., meadows, dwarf shrublands, and tundra; Franklin and Dyrness 1973). The climate of the West Cascade ecoregion broadly consisted of mild summers and cold winters (average minimum to maximum temperature = 1°C–15°C; PRISM 2023) with substantial winter precipitation that varied by elevation (average annual precipitation = 180–300 cm; PRISM 2023), typically consisting of snow above 1200 m. Forests were conifer‐dominant, with common tree species including Douglas fir ( *Pseudotsuga menziesii* ), grand fir ( *Abies grandis* ), mountain hemlock ( *Tsuga mertensiana* ), noble fir ( *A. procera* ), Pacific silver fir ( *A. amabilis* ), subalpine fir ( *A. lasiocarpa* ), western hemlock ( *T. heterophylla* ) and western red cedar ( *Thuja plicata* ).

### 2.2 Data Collection

We conducted scat surveys with detection dog teams in areas of high predicted probability of Sierra Nevada red fox occurrence (> 65%; Quinn et al. 2018), with survey effort focused at high elevations (> 1500 m; Figure 2) typical of montane red fox but not lowland red fox distributions (Aubry 1983; Perrine 2005; Akins 2017). Detection teams consisted of a handler and a dog that was specifically taught, via dehydrated and genetically confirmed scat samples, to detect Sierra Nevada red fox odor (Richards et al. 2021). When surveying for rare species, detection dog enthusiasm and focus are greater if they are rewarded for locating multiple targets (Moriarty et al. 2018), thus the detection dog was additionally taught to alert to scats of secondary carnivore species including American badger, bobcat, coyote, and Pacific marten. Surveys occurred within a randomized and predetermined fixed area (900 ha), typically covering > 15 km linear distance over a minimum search period of 5 h. The detection dog worked off‐leash within visual and vocal recall distance of the handler, searching on‐ and off‐trail as well as in open meadows and on ridgelines (Wasser et al. 2004; Richards et al. 2021). We collected all putative carnivore scats regardless of age or condition. We photographed scats in situ with a ruler for scale, then divided them for collection between a paper bag and a falcon tube (ThermoFisher Scientific, Waltham, Massachusetts, USA) containing > 95% ethanol (8 parts ethanol/1 part scat).

### 2.3 Laboratory Analysis

We used DNA metabarcoding to identify defecator species and vertebrate prey items in collected scats (Eriksson et al. 2019; Massey et al. 2021). We reviewed all previous studies describing montane red fox diet (Aubry 1983; Perrine 2005; Cross and Crabtree 2021; Rosburg‐Francot et al. 2025) to identify putative prey species (Appendix 1 and 2). We extracted DNA in a laboratory dedicated to processing degraded DNA using the DNeasy Blood and Tissue kit (Qiagen, Germantown, Maryland, USA). We included an extraction blank with every batch of extractions as a negative control, where we followed the same extraction protocol but without a scat sample. We amplified a ~ 150 base‐pair DNA segment of the ribosomal mitochondrial 12S gene using a modified version (12S‐V5‐F: GGATTAGATACCCCACTAT; 12S‐V5‐R: TAGAACAGGCTCCTCTAG) of previously published universal vertebrate primers (Riaz et al. 2011). We shifted the 5′ end of 12S‐V5‐F to avoid a 3′ end mismatch (Appendix 1) with pocket gophers (*Thomomys* spp.), which are thought to be important prey items for montane red foxes (Caspi et al. 2025; Rosburg‐Francot et al. 2025). This novel primer also avoids a mismatch near the 3′ end for mountain beaver ( *Aplodontia rufa* ); *in silico* analysis suggested that of potential montane red fox diet items, only amplification of western jumping mouse ( *Zapus princeps* ) might be partially compromised by primer template mismatches, based on one mismatch 4 bp from the 3′ end (Appendix 1). We prepared polymerase chain reactions (PCRs) in triplicate that consisted of 10ul 2× Amplitaq Gold 360 Mastermix (ThermoFisher Scientific, Waltham, Massachusetts, USA), 200 nM each of forward and reverse primer, 2ul of template DNA, and ultrapure water to bring the final volume to 20ul. To aid in identifying contamination from PCR reagents, we included three separate negative control reactions per plate, using ultrapure water as our template (i.e., PCR blanks) in addition to extraction blanks. We amplified each reaction with identical 8‐base pair tags on the 5′ end of the forward and reverse primer that were unique to each sample, to identify individual samples after pooling and prevent misidentification of prey samples due to tag jumping (Schnell et al. 2015; Tosa et al. 2023).

We normalized and pooled PCR products and used the NEBNext Ultra II Library Prep Kit (New England BioLabs, Ipswich, Massachusetts, USA) to add a unique 6‐base pair index and Illumina sequencing adaptors to each library (Illumina Inc., San Diego, California, USA). The first PCR replicate identically tagged forward and reverse primers to prevent the risk of unlabelled amplicons contaminating results and to reduce tag jumping errors. We purified libraries using Solid Phase Reversible Immobilization beads and sent libraries to the Center for Quantitative Life Sciences at Oregon State University for 150‐base pair paired‐end sequencing on the Illumina Nextseq 2000. We paired raw sequence reads using PEAR software (Zhang et al. 2014) and demultiplexed samples based on the 8‐base pair‐index sequences using a custom shell script. We clustered unique sequences at 100% within each sample replicate and assigned taxonomy using BLAST (www.ncbi.nlm.nih.gov/blast) within the LocaTT R package against the 12S sequences in a local database and GenBank curated by MIDORI (Leray et al. 2022). LocaTT provides all equal matches based on bitscore and then filters based on geography to remove species not present regionally (i.e., congeners or confamilials from another part of the world; Goodwin and Levi 2024). With the exception of nuclear mitochondrial pseudogenes, which were diagnosed based on poor percent identification, few reads, and presence with a taxonomically related species with many reads, most sequences had > 99% match. We ran PCR in triplicate and retained all sequences found in at least 2 of 3 replicates with at least 1% relative read abundance, but also produced results with 0.5% relative read abundance to manually examine for any bona fide results that might have been filtered out. We considered scat amplification successful if DNA sequencing produced over 1000 total reads per replicate. We assigned defecator identity to scats from metabarcoding data if: (1) defecator DNA was the only mammalian carnivore DNA detected in the scat, or (2) defecator DNA was one of multiple carnivores identified in the scat, but DNA of other carnivores consisted of < 10% of the total read count for that sample (Tosa et al. 2023). All scats assigned to red fox were collected at elevations > 1400 m, and we assumed that they were of Sierra Nevada red fox origin (e.g., rather than lowland red fox; Lewis et al. 1999; Perrine et al. 2007; Quinn et al. 2018) but did not genetically confirm this.

### 2.4 Diet Patterns and Dietary Overlap

We estimated two measures of occurrence for each prey item to describe Sierra Nevada red fox and coyote diet: (1) frequency of occurrence (FOO), or the proportion of scat samples which contained a given prey item; and (2) weighted percent of occurrence (wPOO), or the proportion a given prey item represented in each scat sample, combined across all samples (Deagle et al. 2019). Frequency of occurrence is a commonly reported metric in analyses of carnivore diets, particularly in studies that use mechanical scat sorting to examine scat contents (Klare et al. 2011; Morin et al. 2019). Although FOO can be informative when describing diet and calculating FOO allowed for comparison between our results and earlier studies, we also calculated wPOO because it provides a better analog for the bioenergetic or biological importance of certain prey, by accounting for the number of taxa within each sample (Tollit et al. 2017; Deagle et al. 2019). Although nonvertebrate food items (e.g., berries, fungi, insects) may be important as secondary, seasonal, or ephemeral resources by carnivores (Newbury and Hodges 2018; Jensen et al. 2022; Smith et al. 2022) including montane red foxes (e.g., Cross and Crabtree 2021; Rosburg‐Francot et al. 2025), we did not have the resources to evaluate the occurrence of nonvertebrate food items in red fox or coyote diet.

We used the “metacoder” R package (Foster et al. 2017) to illustrate phylogenetic patterns of Sierra Nevada red fox and coyote diet and the “iNEXT” R package (Hsieh et al. 2016) to produce a rarefaction curve to estimate completeness and expected taxonomic richness of diet based on sample size. To assess potential for exploitation competition between Sierra Nevada red foxes and coyotes, we calculated dietary overlap using Pianka's overlap index, which ranges from 0 to 1 with a value of 1 indicating complete overlap (Pianka 1973). We estimated mean calculations of Pianka's niche overlap index using the “spaa” R package and referenced these results to a null niche model run in EcoSimR for 10,000 iterations, which produced confidence intervals (CIs) around the Pianka value (Gotelli et al. 2015). The null model randomized the values of diet items consumed by Sierra Nevada red foxes and coyotes while retaining dietary niche width of each species, allowing us to examine whether observed niche overlap differed from null expectations of niche overlap. We calculated overlap using both FOO and wPOO to determine whether estimates of dietary similarity were sensitive to the summary statistic used. We conducted all analyses and produced figures using Program R v 4.1.3 (R Core Team 2021).

## 3 Results

**FIGURE 3:**
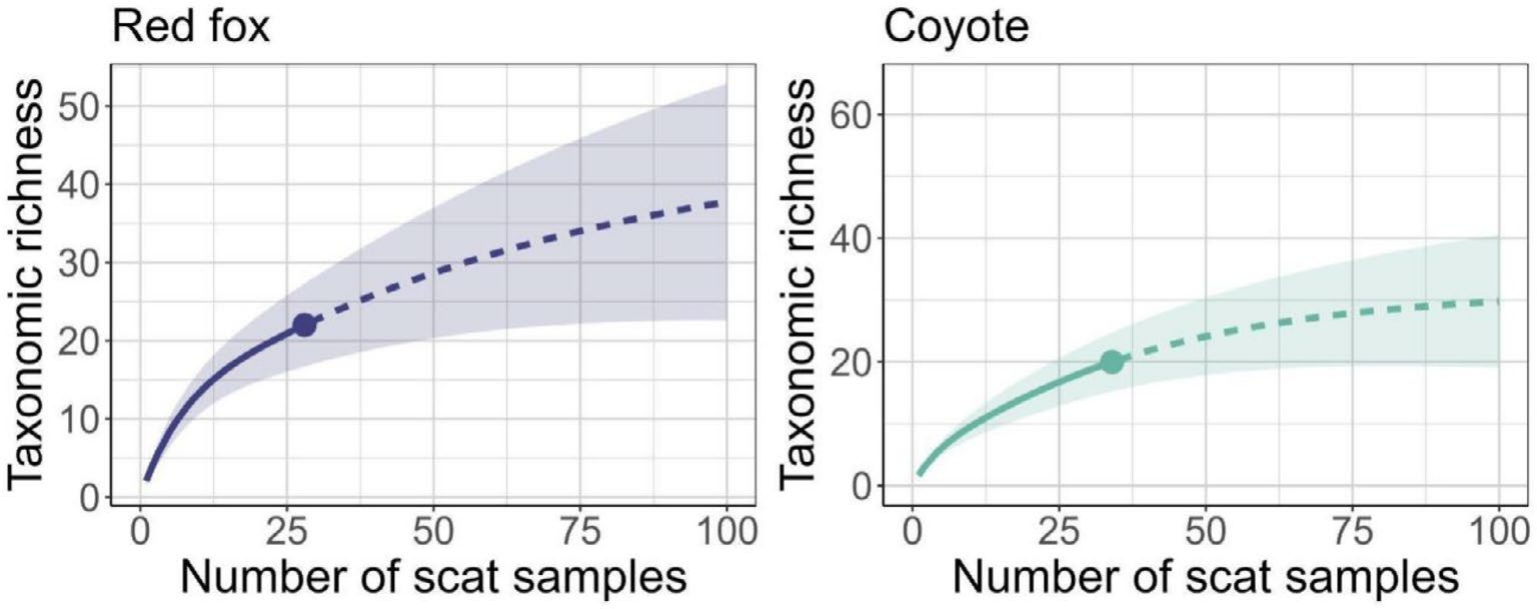
Taxonomic richness of Sierra Nevada red fox ( *Vulpes vulpes necator* ) and coyote ( *Canis latrans* ) diet, based on scats collected during 2017 in the Cascade Mountains of Oregon, USA. The solid portion of the line represents the observed, cumulative species richness from collected samples while the dashed portion of the line represents extrapolated taxonomic richness if additional samples were collected. Shaded areas represent lower and upper 95% confidence intervals from extrapolation.

**FIGURE 4:**
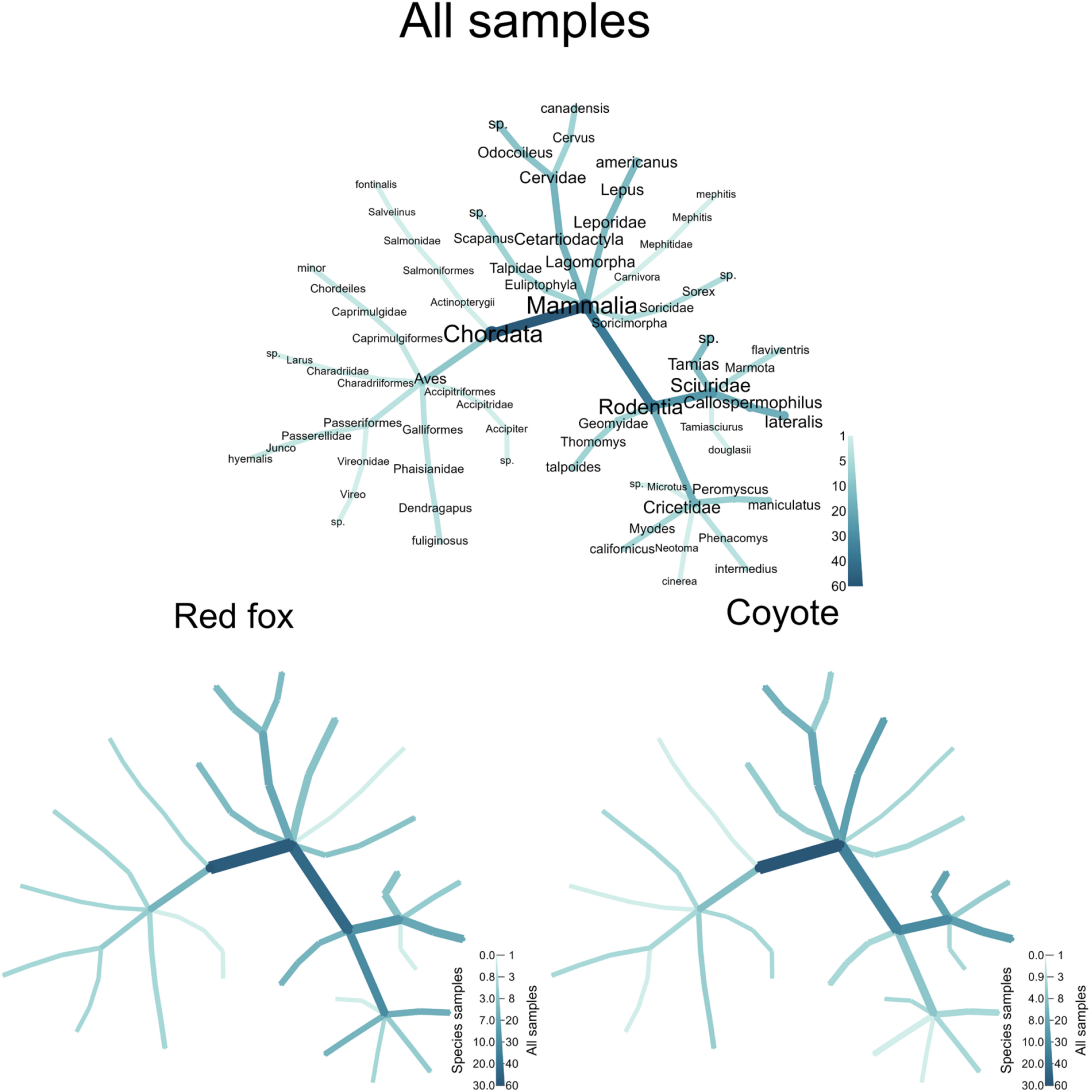
Vertebrate prey items identified via DNA metabarcoding in Sierra Nevada red fox ( *Vulpes vulpes necator* ) and coyote ( *Canis latrans* ) scats collected during 2017 in the Cascade Mountains of Oregon, USA. Taxonomic relationships of prey items are displayed, where color and size of nodes represents the number of samples a given prey item occurred in.

**FIGURE 5:**
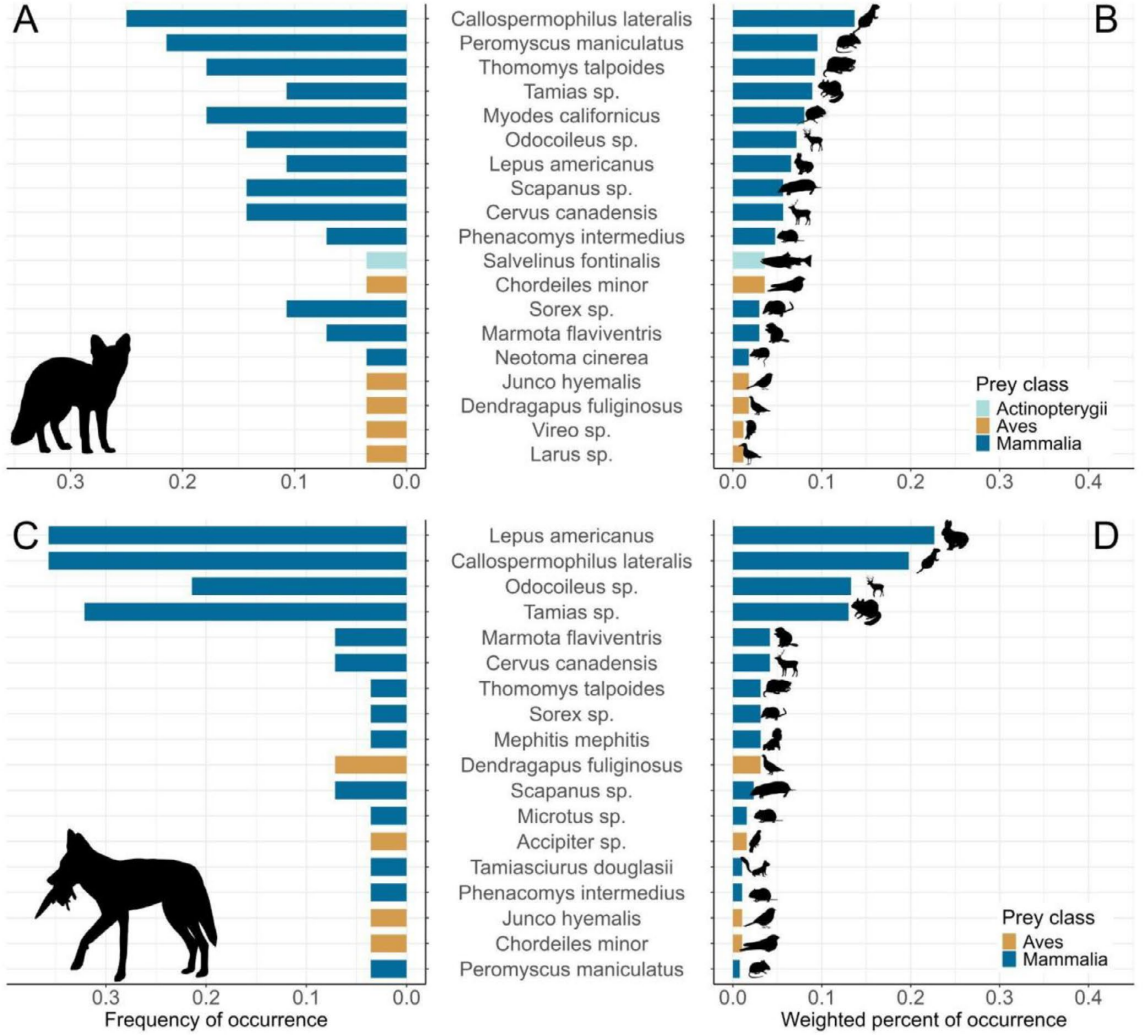
Comparison of vertebrate prey items identified via DNA metabarcoding in Sierra Nevada red fox ( *Vulpes vulpes necator* ) and coyote ( *Canis latrans* ) scats collected during 2017 in the Cascade Mountains of Oregon, USA including: (A) frequency of occurrence (FOO) of items in red fox scats; (B) weighted percent of occurrence (wPOO) of items in red fox scats; (C) FOO of items in coyote scats; and (D) wPOO of items in coyote scats. FOO is the proportion of scat samples which contained a given prey item, whereas wPOO is a weighted measure of the relative importance of prey items. Animal silhouettes were obtained from the R package “phylopic” (Gearty and Jones 2023).

Detection teams surveyed 126.86 km over approximately 10 days in September 2017 and collected 74 carnivore scats (Figure 1, Figure 2). We species‐typed scats as coyote (*n* = 36), Sierra Nevada red fox (*n* = 30), bobcat (*n* = 3), Pacific marten (*n* = 3), and American badger (*n* = 2). We excluded two (6.7%) Sierra Nevada red fox scats and four (11%) coyote scats from further analysis that only contained defecator DNA or only contained diet items that comprised < 1% of reads within the sample, resulting in 28 scat samples to summarize Sierra Nevada red fox diet and 32 scat samples to summarize coyote diet. Estimates of Sierra Nevada red fox and coyote diet overlap varied slightly depending on the summary statistic used to calculate overlap. When using wPOO, Sierra Nevada red fox and coyote diet had higher overlap (Pianka's index = 0.74 [0.53–0.90 CIs]) than predicted by the null model (0.44 [0.27–0.64]). When using FOO to calculate overlap, Sierra Nevada red fox and coyote diet similarly had higher overlap (0.69 [0.50–0.86]) than predicted by the null model (0.47 [0.28–0.65]). Sierra Nevada red foxes and coyotes exhibited similar taxonomic richness in observed diet (Figure 3) and approximately 61% of total prey items identified (*n =* 14 of 23) were consumed by both species (Figure 4, Figure 5).

We identified 19 prey species, 13 prey families, 10 prey orders, and three prey classes in Sierra Nevada red fox scats (Appendix 3). Mammals were the most common prey taxa detected, occurring in 93% (*n* = 25) of scats (Figure 4), while birds were detected in 18% (*n* = 5) of scats. The most frequently occurring prey species (FOO > 0.1) in Sierra Nevada red fox diet were golden‐mantled ground squirrel ( *Callospermophilus lateralis* ), deer mice ( *Peromyscus maniculatus* ), western red‐backed vole ( *Myodes californicus* ), northern pocket gopher ( *T. talpoides* ), elk ( *Cervus canadensis* ), deer (*Odocoileus* sp.), moles (*Scapanus* sp.), snowshoe hare ( *Lepus americanus* ), chipmunks (*Tamias* sp.), and shrews (*Sorex* sp.; Figure 5, Appendix 3). Mammals comprised a larger portion of Sierra Nevada red fox diet (wPOO = 87%) than birds (wPOO = 10%; Figure 5, Appendix 3). Seven mammal species, including golden‐mantled ground squirrel (wPOO = 14%), deer mice (wPOO = 10%), northern pocket gopher (wPOO = 9%), western red‐backed vole (wPOO = 8%), deer (wPOO = 7%), and snowshoe hare (wPOO = 7%) accounted for approximately two‐thirds (63%) of Sierra Nevada red fox diet (Figure 5, Appendix 3). The other 12 species consumed by Sierra Nevada red foxes each had wPOO ≤ 6% and cumulatively represented the remaining 37% of diet (Figure 5, Appendix 3).

We identified 18 prey species, 11 prey families, 10 prey orders, and two prey classes in coyote scats (Appendix 4). Mammals were the most common prey taxa detected, occurring in 100% (*n* = 32) of scats (Figure 4). Additionally, we detected birds in 13% (*n* = 4) of scats. The most frequently occurring prey species (FOO > 0.1) in coyote diet were snowshoe hare, golden‐mantled ground squirrel, chipmunks, and deer (Figure 5, Appendix 4). Mammals comprised a larger portion of coyote diet (wPOO = 95%) than birds (wPOO = 5%; Figure 5, Appendix 4). Four mammal species, including snowshoe hare (wPOO = 23%), golden‐mantled ground squirrel (wPOO = 20%), deer (wPOO = 13%), and chipmunks (wPOO = 13%) accounted for 69% of coyote diet (Figure 5, Appendix 4). The other 14 species consumed by coyotes each had wPOO ≤ 4% and cumulatively represented the remaining 31% of coyote diet (Figure 5, Appendix 4).

## 4 Discussion

Our work provides new information on Sierra Nevada red fox diet in Oregon, a portion of their range for which diet was previously undescribed. Despite a small sample size of scats, Sierra Nevada red foxes in our study had a diverse diet, which would likely have been more diverse (Figure 3) had we been able to collect a larger number of scats via a longer and more comprehensive study (e.g., across different seasons or multiple years). We identified 19 different vertebrate prey items in 30 Sierra Nevada red fox scat samples; in comparison, other montane red fox diet studies have identified 15 vertebrate prey items in 227 samples (Perrine 2005), 20 vertebrate prey items in 413 samples (Aubry 1983), and 50 vertebrate prey items in 296 samples (Rosburg‐Francot et al. 2025). While these apparent discrepancies in dietary diversity could be due to differences in prey species available to each population, it is equally or more plausible that this result is an artifact of scat analysis approaches. Our study and Rosburg‐Francot et al. (2025) used DNA metabarcoding, a more sensitive method for determining prey taxa than other scat analysis methods such as mechanical sorting (Monterroso et al. 2019) which has been used in other montane red fox diet studies (Aubry 1983; Perrine 2005; Cross and Crabtree 2021). Sierra Nevada red fox diet in our study was dominated by small mammals, with rodents and snowshoe hares representing frequently occurring prey items that also comprised a large proportion of consumed prey. Rodents generally appear to be a predominant prey taxon of montane red foxes across populations, comprising 40%–69% FOO in other studies (Aubry 1983; Perrine 2005; Cross and Crabtree 2021; Rosburg‐Francot et al. 2025). Pocket gophers, in particular, are a frequently occurring prey species, representing 18% FOO in our study and 14%–31% FOO elsewhere (Aubry 1983; Perrine 2005; Cross and Crabtree 2021; Rosburg‐Francot et al. 2025).

Some focal prey species or taxa and the importance of other food resources (e.g., carrion) to montane red fox diet appear to vary somewhat among localities. For instance, we identified golden‐mantled ground squirrels as the most frequently occurring prey item (FOO = 28%) of montane red foxes, whereas sciurids have often been secondary prey items in other red fox populations (Perrine 2005; Cross and Crabtree 2021). Snowshoe hares and other large lagomorphs (e.g., white‐tailed jackrabbit [ *Lepus townsendii* ]) were also a frequently occurring prey item in our work (11% FOO) and elsewhere (11%–36% FOO; Cross and Crabtree 2021; Rosburg‐Francot et al. 2025) but were uncommon or essentially absent from other studies (Aubry 1983; Perrine 2005). Cricetids (e.g., voles [*Microtus*, *Myodes*, and *Phenacomys* sp.], deer mice) and similar‐sized species (e.g., shrews, moles [*Scapanus* sp.]) were frequently consumed by most montane red fox populations (20%–53% FOO; Aubry 1983; Cross and Crabtree 2021, *this study*) with one exception (6% FOO; Perrine 2005). Large mammals (e.g., deer, elk) similarly appear to be prominent items in montane red fox diet (16%–35% FOO; Perrine 2005; Cross and Crabtree 2021, *this study*) although not uniformly (2% FOO; Aubry 1983). While a lack of clear dietary patterns among studies putatively supports the premise of montane red foxes as generalist consumers, it is important to acknowledge our relatively small sample size compared to other research efforts (e.g., Aubry 1983; Perrine 2005) when making inferences about red fox diet. In addition, we were unable to evaluate the importance of nonvertebrate foods, which have been indicated as seasonally important in some studies (e.g., whitebark pine [*Pinus albicaulus*] nuts; Cross and Crabtree 2021; Rosburg‐Francot et al. 2025).

Coyote diet was dominated by leporids, rodents, and large mammals, consistent with other studies from the region (Jensen et al. 2022). Although rodents were generally prominent items in coyote diets, smaller‐bodied rodents (e.g., cricetids) were infrequently consumed by coyotes, unlike Sierra Nevada red foxes. Sierra Nevada red fox and coyote diets in our work also differed in the most frequently occurring prey species and prey species that comprised the largest proportions of their diets (golden‐mantled ground squirrel and snowshoe hare, respectively). Despite some dissimilarities in diet, dietary overlap between Sierra Nevada red foxes and coyotes was high and greater than predicted (Figure 4, Figure 5). Golden‐mantled ground squirrels, in particular, were a prevalent prey item in the diet of both species; given that ground squirrels and similar‐sized rodents (e.g., pocket gophers) are common in montane red fox diet elsewhere (Aubry 1983; Perrine 2005; Cross and Crabtree 2021; Rosburg‐Francot et al. 2025), these taxa could represent an important shared resource between foxes and coyotes. Large mammals also appear prominent in both Sierra Nevada red fox and coyote diet, although red foxes are almost certainly scavengers of large mammal carrion, whereas coyotes could be scavenging or predating (Bowen 1981; Gese and Grothe 1995). While scavenging could result in agonistic interactions between montane red foxes and coyotes, it is plausible that coyote‐killed carcasses could also subsidize red fox diets (e.g., Prugh and Sivy 2020; Ruprecht et al. 2021).

Dietary overlap suggests potential for exploitation or interference competition between coyotes and Sierra Nevada red foxes where they occur sympatrically (e.g., Fedriani et al. 2000; Kasper et al. 2016; Vogel et al. 2019; Rosburg‐Francot et al. 2025). Increased sympatry between montane red foxes and coyotes, as well as other presumably dominant carnivores (e.g., bobcats), is expected to occur in the future as these more behaviorally plastic species expand their distributions under changing climatic regimes (Young et al. 2019; Morin et al. 2020; Sirén et al. 2021). Given a narrow ecological niche, the degree to which montane red foxes can adjust to increasing spatial overlap with other carnivores may largely depend on their ability to segregate along other niche axes (e.g., temporal or resource; Manlick et al. 2017; Rodriguez Curras et al. 2022). Although taxonomic richness of Sierra Nevada red fox diet suggests some degree of dietary flexibility, resource niche compression could occur if competition for primary prey species (e.g., golden‐mantled ground squirrel, pocket gophers) increases and red foxes are forced to increase exploitation of items preferred by other carnivores (Randa et al. 2009; Peers et al. 2014; Smith et al. 2016). Future investigations into temporal and spatial variation in montane red fox diet, particularly within the context of diets of other carnivore species, will be valuable for examining the ability of montane red foxes to adapt in an increasingly dynamic world.

DNA metabarcoding provided high‐resolution data on prey species consumed, supporting the emerging utility of this technique compared to more traditional scat analysis methods (e.g., mechanical scat sorting or stable isotope; Kartzinel et al. 2015; Monterroso et al. 2019; Massey et al. 2021). Stable isotope analysis, for example, is useful for broadly quantifying foraging patterns over seasons and years but is generally limited to identifying “functional” prey groups at coarser taxonomic scales (Boecklen et al. 2011). Mechanical scat sorting can similarly underestimate taxonomic richness of carnivore diets, as it is often difficult to distinguish between closely related species and species without nondigestible hard parts may be overlooked (Klare et al. 2011); indeed, up to 10% of mammal remains have gone unidentified in previous montane red fox diet studies (Aubry 1983; Perrine 2005). Nonetheless, DNA metabarcoding methods are not without limitations—issues can arise from reference sample databases (e.g., false‐negative identifications) or laboratory protocols (e.g., primer bias; Elbrecht et al. 2017) and metabarcoding reads are not a perfect analog for biomass consumed (e.g., Shively et al. 2025). Further, metabarcoding reflects a relatively short temporal foraging period (e.g., days) and may be less sensitive to uncommon food items (Cuff et al. 2022). Pairing analytical methods to balance their respective limitations could bolster future efforts (e.g., Hoenig et al. 2022; Nielsen et al. 2018).

Despite conducting field surveys across a large geographic area and focusing sampling within predicted high‐quality habitat (Quinn et al. 2018), Sierra Nevada red fox scats were infrequently collected, highlighting the limitations of our study. For instance, not all red fox and coyote scat collection locations overlapped spatially; thus, it is possible that differences in diet resulted from differential space use rather than differential prey selection. Future studies that are less constrained by temporal or financial considerations and able to collect more scats could examine additional facets of montane red fox diet (e.g., seasonality, nonvertebrate prey consumption). Infrequent scat collection may also underscore the species' rarity; indeed, distribution of the Sierra Nevada red fox has been reduced by approximately two‐thirds since the turn of the 19th century (Green et al. 2023), with an apparently precipitous decline occurring within the last several decades (Perrine et al. 2007; Sacks et al. 2010). Further range contractions of montane red foxes and many other species could occur in coming years, particularly as human‐induced climate change incurs shifts in environmental conditions of montane regions (Beniston 2005; Kulkarni et al. 2013; Wieder et al. 2022). Given that detection dog surveys can be used to noninvasively examine species' presence and are particularly valuable when attempting to detect animals that are uncommon or exist at low densities (Moriarty et al. 2018; Martin et al. 2023), they could offer a powerful tool to identify or clarify areas of montane red fox persistence. We encourage the use of scat surveys with professional detection dog teams as a component of strategies to better elucidate multiple aspects of montane red fox ecology or demography (e.g., population distributions), which can inform fox conservation across the western United States.

## Author Contributions

**Matthew S. Delheimer:** formal analysis (supporting), validation (equal), visualization (equal), writing – original draft (lead). **Marie E. Martin:** formal analysis (equal), validation (equal), visualization (equal), writing – review and editing (equal). **Jennifer Hartman:** conceptualization (equal), funding acquisition (equal), investigation (equal), methodology (equal), project administration (equal), supervision (equal), writing – review and editing (equal). **Katie M. Moriarty:** conceptualization (equal), funding acquisition (equal), investigation (equal), methodology (equal), project administration (equal), supervision (equal), writing – review and editing (equal). **Jennifer M. Allen:** formal analysis (equal), investigation (equal), methodology (equal), writing – review and editing (equal). **Taal Levi:** conceptualization (equal), funding acquisition (equal), investigation (equal), methodology (equal), project administration (equal), supervision (equal), writing – review and editing (equal).

## Ethics Statement

We obtained a scientific taking permit from the Oregon Department of Fish and Wildlife (034‐16, 112‐18) and an Institute for Animal Care and Use Committee waiver from the USDA Forest Service (2017‐005). We worked with professional wildlife detection dog teams.

## Conflicts of Interest

The authors declare no conflicts of interest.

## Data Availability

All required data supporting the findings of this study have been uploaded as Supporting Information and are available in a public repository (https://zenodo.org/records/15642991).

## Appendix 1

Sequence alignments for putative prey species of Sierra Nevada red fox in Oregon, USA based on a review of previous studies describing montane red fox diet (Aubry 1983; Perrine 2005; Cross and Crabtree 2021; Rosburg‐Francot et al. 2025).

## Appendix 2

Putative prey species of Sierra Nevada red fox in Oregon, USA based on a review of previous studies describing montane red fox diet (Aubry 1983; Perrine 2005; Cross and Crabtree 2021; Rosburg‐Francot et al. 2025).

**Table:**

Class	Order	Family	Scientific name	Common name	Montane red fox subspecies
Actinopterygii	Salmoniformes	Salmonidae	*Oncorynchus mykiss*	Rainbow trout	SNRF
Aves				Birds	CRF, RMRF, SNRF
Mammalia	Artiodactyla	Bovidae	*Oreamnos americanus*	Mountain goat	CRF
		Cervidae	*Alces alces*	Moose	RMRF
		Cervidae	*Cervus canadensis*	Elk	CRF, RMRF
		Cervidae	*Odocoileus hemionus*	Mule deer	CRF, RMRF, SNRF
	Carnivora	Mustelidae	*Mustela frenata*	Long‐tailed weasel	CRF
	Euliptophyla	Soricidae	*Sorex* sp.	Shrews	CRF, SNRF
		Talpidae	*Scapanus latimanus*	Broad‐footed mole	SNRF
	Lagomorpha	Leporidae	*Lepus americanus*	Snowshoe hare	CRF, SNRF
			*Lepus* sp.	Hares	RMRF, SNRF
			*Lepus townsendii*	White‐tailed jackrabbit	SNRF
		Ochotonidae	*Ochotona princeps*	American pika	CRF, SNRF
	Rodentia	Aplodontidae	*Aplodontia rufa*	Mountain beaver	SNRF
		Cricetidae	*Cricetidae, Clethrionomys* sp.	Voles	SNRF
			*Clethrionomys gapperi*	Southern red‐backed vole	CRF
			*Microtus montanus*	Montane vole	SNRF
			*Microtus oregoni*	Creeping vole	CRF
			*Microtus richardsoni*	North American water vole	CRF
			*Neotoma cinerea*	Bushy‐tailed woodrat	CRF, RMRF
			*Ondatra zibethicus*	Muskrat	SNRF
			*Peromyscus maniculatus*	Deer mouse	CRF, SNRF
			*Peromyscus* sp.	Mice	SNRF
			*Phenacomys intermedius*	Western heather vole	CRF
			*Reithrodontomys megalotis*	Western harvest mouse	SNRF
		Geomyidae	*Thomomys monticola*	Mountain pocket gopher	SNRF
			*Thomomys talpoides*	Northern pocket gopher	CRF, RMRF
		Sciuridae	*Callospermophilus lateralis*	Golden‐mantled ground squirrel	SNRF
			*Glaucomys sabrinus*	Northern flying squirrel	CRF
			*Marmota caligata*	Hoary marmot	CRF
			*Marmota flaviventris*	Yellow‐bellied marmot	RMRF, SNRF
			*Spermophilus saturatus*	Cascade golden‐mantled ground squirrel	CRF
			*Spermophilus* sp.	Ground squirrels	RMRF, SNRF
			*Tamias* sp.	Chipmunks	RMRF, SNRF
			*Tamias townsendii*	Townsend's chipmunk	CRF
			*Tamiasciurus douglasii*	Douglas' squirrel	SNRF
			*Tamiasciurus hudsonicus*	Red squirrel	RMRF
		Zapodidae	*Zapus princeps*	Western jumping mouse	SNRF
			*Zapus trinotatus*	Pacific jumping mouse	CRF

Abbreviations: CRF, Cascades red fox; RMRF, Rocky Mountain red fox; SNRF, Sierra Nevada red fox.

## Appendix 3

Occurrence of vertebrate prey items in Sierra Nevada red fox ( *Vulpes vulpes necator* ) scats collected during 2017 in Oregon, USA. We report the number of samples (*n*), frequency of occurrence (FOO), and weighted percent of occurrence (wPOO) for each prey item.

**Table:**

Class	Order	Family	Scientific name	Common name	*n*	FOO	wPOO
Actinopterygii	Salmoniformes	Salmonidae	*Salvelinus fontinalis*	Brook trout	1	0.04	0.04
Aves	Caprimulgiformes	Caprimulgidae	*Chordeiles minor*	Common nighthawk	1	0.04	0.04
	Charadriiformes	Charadriidae	*Larus* sp.	Gull	1	0.04	0.01
	Galliformes	Phaisianidae	*Dendragapus fuliginosus*	Sooty grouse	1	0.04	0.02
	Passeriformes	Passerellidae	*Junco hyemalis*	Dark‐eyed junco	1	0.04	0.02
		Vireonidae	*Vireo* sp.	Vireo	1	0.04	0.01
Mammalia	Cetartiodactyla	Cervidae	*Cervus canadensis*	Elk	4	0.14	0.06
			*Odocoileus* sp.	Deer	4	0.14	0.07
	Euliptophyla	Talpidae	*Scapanus* sp.	Mole	4	0.14	0.06
	Lagomorpha	Leporidae	*Lepus americanus*	Snowshoe hare	3	0.11	0.07
	Rodentia	Cricetidae	*Myodes californicus*	Western red‐backed vole	5	0.18	0.08
			*Neotoma cinerea*	Dusky‐footed woodrat	1	0.04	0.02
			*Peromyscus maniculatus*	Deer mouse	6	0.21	0.10
			*Phenacomys intermedius*	Western heather vole	2	0.07	0.05
		Geomyidae	*Thomomys talpoides*	Northern pocket gopher	5	0.18	0.09
		Sciuridae	*Callospermophilus lateralis*	Golden‐mantled ground squirrel	7	0.25	0.14
			*Marmota flaviventris*	Yellow‐bellied marmot	2	0.07	0.03
			*Tamias* sp.	Chipmunk	3	0.11	0.09
	Soricomorpha	Soricidae	*Sorex* sp.	Shrews	3	0.11	0.03

## Appendix 4

Occurrence of vertebrate prey items in coyote ( *Canis latrans* ) scats collected during 2017 in Oregon, USA. We report number of samples (*n*), frequency of occurrence (FOO), and weighted percent of occurrence (wPOO) for each prey item.

**Table:**

Class	Order	Family	Scientific name	Common name	*n*	FOO	wPOO
Aves	Accipitriformes	Accipitridae	*Accipiter* sp.	Accipiter	1	0.04	0.02
	Caprimulgiformes	Caprimulgidae	*Chordeiles minor*	Common nighthawk	1	0.04	0.01
	Galliformes	Phaisianidae	*Dendragapus fuliginosus*	Sooty grouse	2	0.07	0.03
	Passeriformes	Passerellidae	*Junco hyemalis*	Dark‐eyed junco	1	0.04	0.01
Mammalia	Carnivora	Mephitidae	*Mephitis mephitis*	Striped skunk	1	0.04	0.03
	Cetartiodactyla	Cervidae	*Cervus canadensis*	Elk	2	0.07	0.04
			*Odocoileus* sp.	Deer	6	0.21	0.13
	Euliptophyla	Talpidae	*Scapanus* sp.	Mole	2	0.07	0.02
	Lagomorpha	Leporidae	*Lepus americanus*	Snowshoe hare	10	0.36	0.23
	Rodentia	Cricetidae	*Microtus* sp.	Vole species	1	0.04	0.02
			*Peromyscus maniculatus*	Deer mouse	1	0.04	0.01
			*Phenacomys intermedius*	Western heather vole	1	0.04	0.01
		Geomyidae	*Thomomys talpoides*	Northern pocket gopher	1	0.04	0.03
		Sciuridae	*Callospermophilus lateralis*	Golden‐mantled ground squirrel	10	0.36	0.20
			*Marmota flaviventris*	Yellow‐bellied marmot	2	0.07	0.04
			*Tamias* sp.	Chipmunk	9	0.32	0.13
			*Tamiasciurus douglasii*	Douglas' squirrel	1	0.04	0.01
	Soricimorpha	Soricidae	*Sorex* sp.	Shrews	1	0.04	0.03
